# Phylogenetic analysis of Newcastle disease viruses isolated from waterfowl in the Upper Midwest Region of the United States

**DOI:** 10.1186/1743-422X-6-191

**Published:** 2009-11-05

**Authors:** Naresh Jindal, Yogesh Chander, Ashok K Chockalingam, Martha de Abin, Patrick T Redig, Sagar M Goyal

**Affiliations:** 1 Department of Veterinary Population Medicine, College of Veterinary Medicine, University of Minnesota, 1333 Gortner Avenue, Saint Paul, MN, 55108, USA

## Abstract

**Background:**

This study was conducted to characterize Newcastle disease virus (NDV) isolates obtained from waterfowl from the Upper Midwest region of the United States. A total of 43 NDVs were isolated by inoculation of cloacal samples in embryonated chicken eggs. These isolates were obtained from 24 mallards, seven American green-winged teals, six northern pintails, four blue-winged teals, and two wood ducks. Partial sequences of fusion gene were analyzed to determine the pathotypes and genotypes involved.

**Results:**

Deduced amino acid sequence of the cleavage site of fusion (F) protein revealed that all isolates had avirulent motifs. Of the 43 isolates, 23 exhibited sequence motif of ^111^GGKQGRL^117 ^at the cleavage site, 19 exhibited ^111^GEKQGRL^117 ^while one isolate showed ^111^GERQGRL^117^. Phylogenetic analysis based on comparison with different classes of NDVs revealed that all 43 isolates clustered with class II NDVs and none with class I NDVs. Within class II, five isolates were phylogenetically close to genotype I NDVs while the remaining 38 were close to genotype II.

**Conclusion:**

We conclude that more than one genotype of NDV circulates in waterfowl in the Upper Midwest region of the US. Continuous surveillance may help better understand the epidemiology of NDVs maintained in wild bird populations and their relationship to NDVs in domestic poultry, if any.

## Background

Avian paramyxoviruses (APMV) belong to genus *Avulavirus *in the family *Paramyxoviridae*. The genome of APMV is an approximately 15 kb long, negative-sense, single-stranded RNA molecule. It has six genes that encode for a nucleoprotein (N), a phosphoprotein (P), a matrix protein (M), a fusion protein (F), an attachment protein called hemagglutinin-neuraminidase (HN), and a large polymerase protein (L) [1]. Nine serotypes of avian paramyxoviruses (APMV-1 to APMV-9) have been identified. Of these, APMV-1, also called the Newcastle disease virus (NDV), is the causative agent of Newcastle disease (ND) in poultry. Based on genetic and antigenic analyses of NDV isolates, two major classes (class I and class II) are identified [2,3] and each class has nine genotypes (1-9 genotypes in class I and I-IX in class II) [4,5].

The NDV can cause clinical signs varying from subclinical infections to 100% mortality, depending on the susceptibility of the host and the virulence of the virus. The virus is categorized into velogenic (velogenic neurotropic or velogenic viscerotropic), mesogenic, lentogenic, and asymptomatic enteric strains on the basis of their pathogenesis and virulence. The velogenic strains cause acute fatal infection of chickens of all age groups with clinical findings of nervous signs or extensive hemorrhagic lesions in the gastrointestinal tract. The mesogenic strains are of intermediate virulence and cause moderate respiratory signs with occasional nervous signs while the lentogenic strains cause mild to inapparent infections [1]. The lentogenic strains have been detected in both domestic poultry [6-8] and wild bird populations [4,8,9]. Though velogenic strains are considered exotic (exotic Newcastle disease, END) to US poultry, these strains have been isolated occasionally from different avian species in the US [10,11]. During 2002-2003, California outbreak of END in backyard fowl and commercial poultry resulted in the destruction of about 3.3 million birds and cost $200 million dollars to control the disease [11,12]. Outbreaks of ND have been reported in many countries with considerable economic losses [1]. Such outbreaks warrant continuous surveillance for END in commercial poultry and wild birds.

The surveillance of NDVs in waterfowl is sporadic and often occurs with other monitoring programs such as those for avian influenza viruses (AIV) [13,14]. Wild birds are considered the natural reservoirs of NDVs and mostly harbor lentogenic strains. Studies on genetic diversity among lentogenic strains of NDVs revealed that some of the NDVs from waterfowl and shorebirds were phylogenetically related with NDVs isolated from live-bird markets in the US [4]. It is recommended that epidemiological studies should be continued to determine the prevalence of lentogenic NDVs in wild bird populations [4]. An epidemiological link between isolates recovered from outbreaks in domestic poultry with those obtained from wild bird populations has also been suggested [8,9,15,16]. Therefore, continuous surveillance of wild bird populations may help better understand the NDVs circulating in the environment. This study was conducted to characterize NDV isolates obtained from waterfowl samples. In this study, the cloacal samples from waterfowl from Upper Midwest region of the US were initially screened for AIV by real time reverse transcription-polymerase chain reaction (rRT-PCR); the AIV positive samples by rRT-PCR were inoculated on to the embryonated eggs for virus isolation that yielded NDV in some of them. The NDV isolates were characterized by sequencing to determine the pathotypes and genotypes involved and the changes at the nucleotide and amino acid levels.

## Results

Altogether, 159 viral isolations from cloacal samples of AIV rRT-PCR-positive waterfowl (n = 890) were obtained, as shown by hemagglutinating (HA) activity of allantoic fluid in embryonated eggs. Of these, 43 were positive for NDV by reverse transcription-polymerase chain reaction (RT-PCR). BLAST analysis of partial sequences of F gene of NDV isolates confirmed their identity. These isolates were obtained from 24 mallards (MALL; *Anas platyrhynchos*), seven American green-winged teals (AGWT; *Anas crecca*), six northern pintails (NOPI; *Anas acuta*), four blue-winged teals (BWTE; *Anas discors*), and two wood ducks (WODU; *Aix sponsa*). Spatial distribution revealed that 28 isolates were obtained from South Dakota, 14 from Minnesota, and 1 from North Dakota.

### Cleavage site analysis

The F gene portion (333 base pairs) corresponding to nucleotide positions 170-502 of GenBank accession number AF217084 was sequenced. Deduced amino acid sequences of the F gene cleavage site were used to determine the pathotypes involved and are shown in Table 1. The fusion gene of virulent NDVs is characterized by the presence of a pair of dibasic amino acids at the cleavage site while in lentogenic strains it is characterized by the presence of monobasic amino acids. None of the isolates had the sequence motif of ^111^GR/KRQRK/RF^117^, a characteristic of the virulent strains. All 43 NDVs had an avirulent motif of monobasic amino acids at their F gene cleavage sites. Of the 43 isolates, 23 exhibited sequence motif of ^111^GGKQGRL^117^, 19 exhibited the sequence motif of ^111^GEKQGRL^117^, and one isolate exhibited the sequence motif of ^111^GERQGRL^117 ^at the cleavage site of F gene.

**Table 1:** Details of Newcastle disease viral isolates of this study.

**GenBank** **accession** **number**	**Isolate name**	**Fusion gene** **cleavage** **site (111-117)**	**Class**	**Genotype**	**Species**	**State**	**Country**
GQ229531	NDV-001/US(MN)/2008	GEKQGRL	II	II	Mallard	Minnesota	USA
GQ229532	NDV-002/US(MN)/2008	GGKQGRL	II	I	AGWT^A^	Minnesota	USA
GQ229533	NDV-003/US(MN)/2008	GEKQGRL	II	II	AGWT	Minnesota	USA
GQ229534	NDV-004/US(MN)/2008	GEKQGRL	II	II	Mallard	Minnesota	USA
GQ229535	NDV-006/US(MN)/2008	GGKQGRL	II	II	Northern pintail	Minnesota	USA
GQ229536	NDV-007/US(SD)/2008	GGKQGRL	II	I	Northern pintail	South Dakota	USA
GQ229537	NDV-009/US(SD)/2008	GEKQGRL	II	II	Mallard	South Dakota	USA
GQ229538	NDV-011/US(SD)/2008	GGKQGRL	II	I	Mallard	South Dakota	USA
GQ229539	NDV-012/US(SD)/2008	GGKQGRL	II	II	Mallard	South Dakota	USA
GQ229540	NDV-013/US(SD)/2008	GGKQGRL	II	II	Mallard	South Dakota	USA
GQ229541	NDV-015/US(SD)/2008	GEKQGRL	II	II	Northern pintail	South Dakota	USA
GQ229542	NDV-016/US(SD)/2008	GEKQGRL	II	II	Mallard	South Dakota	USA
GQ229543	NDV-017/US(SD)/2008	GEKQGRL	II	II	Mallard	South Dakota	USA
GQ229544	NDV-018/US(SD)/2008	GEKQGRL	II	II	Mallard	South Dakota	USA
GQ229545	NDV-019/US(SD)/2008	GGKQGRL	II	II	Mallard	South Dakota	USA
GQ229546	NDV-020/US(SD)/2008	GGKQGRL	II	II	Mallard	South Dakota	USA
GQ229547	NDV-021/US(SD)/2008	GGKQGRL	II	II	Mallard	South Dakota	USA
GQ229548	NDV-022/US(SD)/2008	GGKQGRL	II	II	AGWT	South Dakota	USA
GQ229549	NDV-023/US(SD)/2008	GGKQGRL	II	II	Mallard	South Dakota	USA
GQ229550	NDV-024/US(SD)/2008	GGKQGRL	II	I	Northern pintail	South Dakota	USA
GQ229551	NDV-025/US(SD)/2008	GGKQGRL	II	II	AGWT	South Dakota	USA
GQ229552	NDV-026/US(SD)/2008	GEKQGRL	II	II	Northern pintail	South Dakota	USA
GQ229553	NDV-027/US(SD)/2008	GEKQGRL	II	II	Mallard	South Dakota	USA
GQ229554	NDV-028/US(SD)/2008	GEKQGRL	II	II	Mallard	South Dakota	USA
GQ229555	NDV-029/US(SD)/2008	GEKQGRL	II	II	Mallard	South Dakota	USA
GQ229556	NDV-030/US(SD)/2008	GEKQGRL	II	II	Mallard	South Dakota	USA
GQ229557	NDV-031/US(SD)/2008	GEKQGRL	II	II	Mallard	South Dakota	USA
GQ229558	NDV-032/US(SD)/2008	GEKQGRL	II	II	Mallard	South Dakota	USA
GQ229559	NDV-033/US(SD)/2008	GEKQGRL	II	II	Mallard	South Dakota	USA
GQ229560	NDV-034/US(SD)/2008	GEKQGRL	II	II	Mallard	South Dakota	USA
GQ229561	NDV-035/US(SD)/2008	GEKQGRL	II	II	AGWT	South Dakota	USA
GQ229562	NDV-036/US(MN)/2008	GGKQGRL	II	II	Wood duck	Minnesota	USA
GQ229563	NDV-037/US(MN)/2008	GGKQGRL	II	II	Mallard	Minnesota	USA
GQ229564	NDV-038/US(MN)/2008	GGKQGRL	II	II	Blue-winged teal	Minnesota	USA
GQ229565	NDV-039/US(MN)/2008	GGKQGRL	II	II	Blue-winged teal	Minnesota	USA
GQ229566	NDV-040/US(MN)/2008	GGKQGRL	II	II	AGWT	Minnesota	USA
GQ229567	NDV-041/US(MN)/2008	GGKQGRL	II	II	AGWT	Minnesota	USA
GQ229568	NDV-042/US(MN)/2008	GGKQGRL	II	II	Blue-winged teal	Minnesota	USA
GQ229569	NDV-043/US(MN)/2008	GGKQGRL	II	II	Wood duck	Minnesota	USA
GQ229570	NDV-048/US(SD)/2008	GGKQGRL	II	II	Blue-winged teal	South Dakota	USA
GQ229571	NDV-049 US(MN)/2008	GERQGRL	II	I	Mallard	Minnesota	USA
GQ229572	NDV-050/US(SD)/2008	GEKQGRL	II	II	Northern pintail	South Dakota	USA
GQ229573	NDV-051/US(ND)/2008	GGKQGRL	II	II	Mallard	North Dakota	USA

^A^AGWT = American Green-winged teal

### Phylogenetic analysis

**Figure 1:**
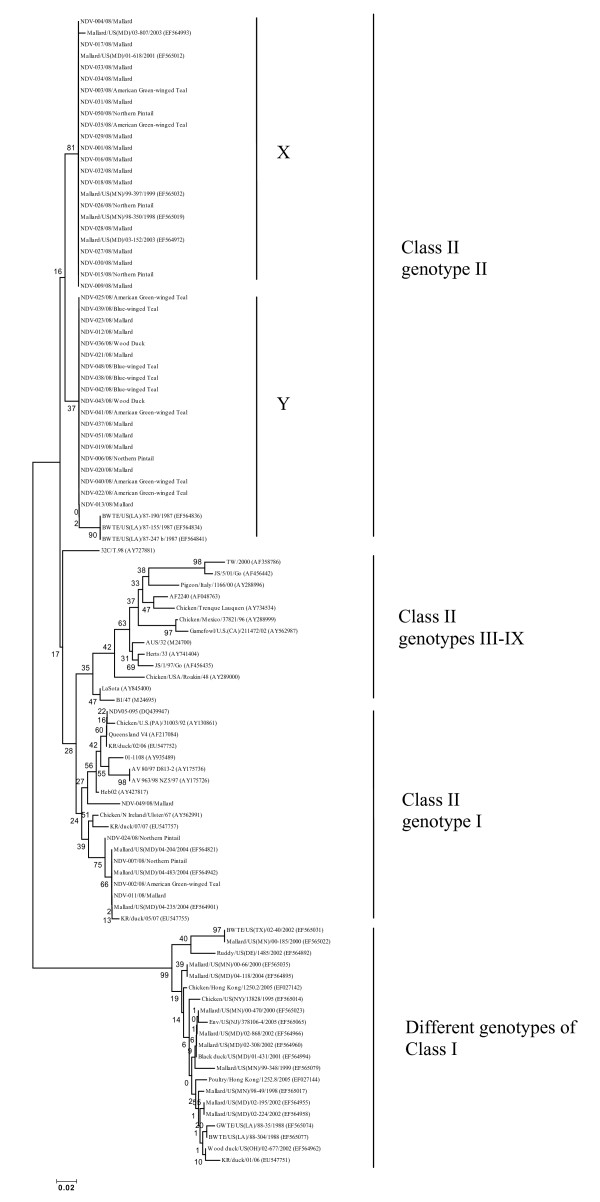
**Phylogenetic tree based on partial nucleotide sequences [corresponding to nucleotide positions 170-502 of **GenBank: AF217084**] of fusion gene of Newcastle disease virus**. The sequences starting with NDV (without accession numbers) are from the present study, and the sequences with virus name (GenBank accession numbers) are previously published sequences of NDVs. The phylogenetic tree was constructed by Neighbor-Joining method, 500 bootstrap replicates (bootstrap values are shown on tree).

Phylogenetic analysis of partial F gene nucleotide sequences of NDV isolates was done by comparing them with already published F gene sequences of both class I and class II NDVs. None of the isolates clustered with class I NDVs (Figure 1); all isolates clustered with class II NDVs (Figure 1). Within class II, all isolates clustered with genotype I or II. Five of the 43 isolates clustered with NDV sequences of genotype I/Ia suggesting them to belong to genotype I (Figure 1). Four of the five isolates clustered together with genotype I NDVs from the US [Mallard/US(MD)/04-483/2004, EF564942; Mallard/US(MD)/04-204/2004, EF564821; and Mallard/US(MD)/04-235/2004, EF564901] and Korea [KR/duck/05/07, EU547755]. The sequence homology among these four isolates was 99.6% to 100% at the nucleotide level. The remaining one isolate was in a different group from these four isolates and was phylogenetically closer to genotype I NDVs from China [Heb02, AY427817], the US [AV 80/97 D813-2, AY175736] and Ireland [AV 963/98 NZ5/97, AY175726]. This isolate had sequence homology of 90.9% to 90.4% at nucleotide level with the other four isolates of genotype I of this study. All five genotype I isolates had sequence homology of 87.9% to 100% with class II genotype I NDVs used for comparison.

The remaining 38 isolates clustered with genotype II NDVs. These isolates clustered into two groups with 19 isolates in each group. For ease of understanding, we have named these two groups as X and Y (Figure 1). The isolates in group X were phylogenetically close to genotype IIa NDVs from wild birds from different regions of the US [Mallard/US(MD)/03-152/2003, EF564972; Mallard/US(MD)/01-618/2001, EF565012; Mallard/US(MN)/99-397/1999, EF565032; Mallard/US(MN)/98-350/1998, EF565019; and Mallard/US(MD)/03-807/2003, EF564993]. The isolates in group X were also phylogenetically close to a genotype IIa NDV from Argentina [32C/T.98, AY727881], but the latter was in a different group. None of the already reported NDV sequences of class II genotype II used for comparison clustered together with NDV isolates of group Y. Though the isolates in group Y were phylogenetically close to already reported genotype IIa NDVs from wild birds in the US [Blue winged teal/US(LA)/87-190/1987, EF564836; Blue winged teal/US(LA)/87-155/1987, EF564834; Blue winged teal/US(LA)/87-247_b/1987, EF564841], they were not in the same group. The vaccine strains [LaSota, AY845400; B1, M24695] clustered in a different group from isolates of this study. All already published sequences of velogenic strains with in class II were phylogenetically distinct from NDVs of this study (Figure 1). The sequence homology of genotype II isolates of this study ranged from 95.5% to 100% at the nucleotide level, and the homology as compared to already published sequences of class II genotype II ranged from 90.4% to 100%.

## Discussion

This study was conducted to characterize NDVs isolated from waterfowl in the Upper Midwest region of the US. The initial aim of this study was to isolate and characterize AIV from waterfowl. During the study period, 7458 cloacal samples were collected and of these, 11.9% samples were AIV positive by rRT-PCR. Inoculation of these AIV positive samples in embryonated chicken eggs yielded hemagglutinating viruses and of these, 43 were identified as NDVs by RT-PCR using primer specific for F gene. We were expecting the isolation of AIV rather than NDV on inoculation in embryonated eggs as the samples were initially positive for AIV by rRT-PCR. The possibility of the presence of other hemagglutinating virus(es) in HA positive-AIV negative (by RT-PCR for matrix gene)-NDV negative (by RT-PCR for F gene) allantoic fluid cannot be ruled out and testing of such allantoic fluid is underway in our laboratory. The isolation of NDV from samples that were rRT-PCR positive for AIV indicates that the cloacal sample may have mixed infection with NDV and AIV with concentration of NDV being higher than that of AIV. Hence, the NDV probably overgrew AIV upon inoculation in embryonated chicken eggs. It is to be noted that we tested only AI rRT-PCR positive samples by inoculation in embryonated eggs; testing of more samples might have led to isolation of more NDVs. The isolation of NDV from AIV positive samples indicates the presence of both viruses (AIV and NDV) in waterfowl. The AIV positive allantoic fluid by RT-PCR was not tested for NDV; this testing might provide a better picture of mixed infection of both NDV and AIV. Mixed infection of AIV and NDV in waterfowl has been reported earlier [17,18].

A large amount of sequence data on NDVs isolated throughout the world has been published over the years and is now available for sequence comparison and phylogenetic analysis which can be used to predict the pathotypes and to determine the origin of NDV outbreaks. It has been well established that cleavage of NDV fusion protein is a major determinant for viral virulence. In this study, the F gene sequence of NDVs was used for pathotyping as well as their characterization into different classes and genotypes. None of the isolates was found to be velogenic on the basis of sequence motif of F gene cleavage site. It has been reported that virulent virus has at least one pair of basic amino acids at residues 115 and 116 plus a phenylalanine at residue 117 and a basic amino acid (R) at 113 at the cleavage site whereas lentogenic strains lack dibasic amino acids [19]. All NDV isolates of this study had lentogenic motif at the cleavage site. These results are in agreement with previous studies reporting the detection of lentogenic NDVs in wild birds and domestic ducks [4,9,15,20,21]. None of the isolates had the sequence motif of ^111^GERQE/DRL^117 ^of class I isolates, although the latter have been reported in wild birds and domestic ducks [4,21]. For example, [4] reported seven of the nine genotypes of class I NDVs in waterfowl and shore birds in the US while [21] reported the presence of class I genotype 2 NDVs in domestic ducks in Korea.

Of the 43 isolates, 42 had the sequence motif of ^111^GG/EKQGRL^117 ^at the cleavage site and were phylogenetically similar to either genotype I or genotype II within class II. This sequence motif has been reported earlier in genotypes I and II of class II NDVs [4]. However, a different sequence motif (^111^GRRQRRF^117^) was reported in the lentogenic strains from Australia [22]. One of the isolates had the sequence motif of ^111^GERQGRL^117 ^and this isolate also clustered with class II genotype I strains. This isolate differed from other 42 isolates in the sense that the amino acid lysine was replaced by arginine at position 113.

Overall genotype II viruses were more predominant than genotype I viruses in this study. This finding has the support of [4] who also observed more genotype IIa viruses than genotype I viruses within class II. The NDV isolates in this study were derived only from rRT-PCR AIV positive samples, the possibility of presence of genotypes of both classes (that were not detected in this study) in rRT-PCR AIV negative samples cannot be ruled out. Within class II, the NDV sequences clustered into two different groups. None of the isolates was phylogenetically close to vaccine strains used for comparison. This indicates that in spite of the regular use of live vaccines in poultry throughout the world, their transmission to wild birds may not be a common phenomenon. In an earlier study, [4] also did not detect any vaccine strains in wild birds in the US. Since wild birds have been reported to be a reservoir of NDV [16,23], the mixing of different species at stop-overs during migration and the sharing of common wintering and breeding areas may provide opportunity for virus spread within and between countries and may help perpetuate different genotypes and classes of NDVs in these birds. The phylogenetic proximity of our isolates with those from the US, China, Korea, and Ireland points to this likelihood.

The presence of class II viruses in wild birds is of concern because this class of viruses has been responsible for several panzootics of Newcastle disease in poultry [24,25]. There are reports suggesting that velogenic NDVs might arise from lentogenic NDVs in nature [23,26]. Further, studies have also suggested that point mutation, and not gene recombination, may be responsible for generating virulent and avirulent strains. For example, the NDV outbreak in Australian poultry during 1998-2000 was caused by a virulent NDV that originated due to mutation in a class II genotype I virus [26]. These authors were of the opinion that lentogenic viruses have the potential to become virulent with the passage of time. Even passaging of NDVs from one host to another has been reported to increase their virulence [16,27]. In addition, the selective forces imposed by a new host environment may also play a role in acquisition of virulence [28]. These findings suggest that the lentogenic strains from wild birds may acquire virulence by waterfowl-to-domestic poultry transmission in nature. In such a scenario we may encounter an NDV outbreak in domestic poultry.

Similar to low pathogenic AIV, the lentogenic NDVs in wild bird populations invariably do not cause obvious disease. Even virulent strains of NDVs that are lethal to chickens, have been isolated from apparently healthy domestic ducks [14,29,30]. Though virulent strains of NDVs were not detected in this study, their presence in the population cannot be ruled out in view of the potential created by the comingling nature and migration patterns of wild birds within and across continents. Thus, continuous surveillance for NDV in wild birds is essential for better understanding of its epidemiology. In conclusion, the present study reveals the circulation of class II (genotypes I and II) lentogenic strains of NDVs in wild birds in the Upper Midwest region of the US. Further studies are needed to determine the true prevalence and implications of various genotypes of NDV within wild bird population.

## Conclusion

This study indicates the circulation of class II genotypes I and II NDVs in waterfowl in the Upper Midwest region of the US with an avirulent motif of monobasic amino acids at their F gene cleavage sites. Phylogenetically distant relationship of NDVs of this study with vaccine strains indicates that in spite of the regular use of live vaccines in poultry, their transmission to wild birds may not be a common phenomenon.

## Methods

### Sample collection

Under an NIH funded surveillance program on avian influenza, cloacal and oropharyngeal (OP) swabs were collected from various waterfowl species in Minnesota, South Dakota, and North Dakota from April 2008 to October 2008. The swabs were placed in brain heart infusion broth containing antibiotics (penicillin 500 IU/mL, streptomycin 500 μg/mL, neomycin 0.15 mg/mL, fungizone 1.5 μg/mL, and gentamicin 50 μg/mL) and were transported on ice to the laboratory. The initial aim of the project was to test cloacal samples (n = 7458) from waterfowl species for the detection of AIV for which five samples each were pooled and the pools were tested for AIV using rRT-PCR [31]. Individual samples in positive pools were then tested for the detection of AIV by rRT-PCR.

### Virus isolation

Individual samples positive for AIV by rRT-PCR (n = 890) were inoculated in 9-day-old specific pathogen free embryonated chicken eggs for virus isolation (VI). Allantoic fluid from inoculated eggs was harvested four days post inoculation and subsequently tested for hemagglutination (HA) using 0.5% turkey erythrocytes. The HA positive allantoic fluids (n = 159) were tested by RT-PCR for the confirmation of AIV as described below.

### Total RNA extraction and RT-PCR

Total RNA was extracted from allantoic fluids and a known AIV isolate using QIAamp Viral RNA extraction kit (Qiagen, Valencia, CA). Extracted RNAs were subjected to RT-PCR using primers targeting the matrix gene of AIV [32]. A band of 1027 base pairs was observed in 52 cases indicating them to be AIV. The HA positive allantoic fluids that were negative for AIV (n = 107) were then tested for NDV by RT-PCR. Total RNA extracted from a known APMV-1 was used as a positive control. The RNA was amplified using primers specific to the F gene of NDV [33]. PCR amplification was carried out using Qiagen OneStep RT-PCR kit (Qiagen, Valencia, CA). Amplified PCR products were electrophoresed on 1.2% agarose gel. A band of 356 base pairs was observed in 43 cases indicating them to be NDVs. Further studies are underway to determine the identity of the remaining HA positive allantoic fluids (n = 64). The NDV positive PCR products were purified using a PCR purification kit (Qiagen, Valencia, CA) and were then sequenced in both directions at the BioMedical Genomic Center, University of Minnesota.

### Phylogenetic analysis

The forward and reverse nucleotide sequences of all 43 isolates were curated, edited and aligned using a "Sequencher" software . The aligned sequences were analyzed on NCBI website  using BLAST to confirm their identity. The nucleotide sequences were then aligned using MEGA 4.0 software by Clustal W method. The evolutionary distances were computed by Pairwise Distance method using the Maximum Composite Likelihood Model. A phylogenetic tree of aligned sequences was constructed by Neighbor-Joining method (500 replicates for bootstrap). The F gene nucleotide sequences [corresponding to nucleotide positions 170-502 of GenBank: AF217084] were translated to deduced amino acid sequences to determine the pathotype involved. The nucleotide sequences were also compared with NDV sequences available in the GenBank. The virus types and their GenBank accession numbers used for comparison are given in Tables 2 and 3. These included F gene sequences of different genotypes of class I and class II NDVs. To maintain uniformity and consistency, class I genotypes are indicated using Arabic numerals (1-9) while class II genotypes are indicated using Roman numerals (I-IX).

**Table 2:** Previously published F gene sequences of class I Newcastle disease virus used for phylogenetic analysis.

**GenBank** **accession** **number**	**Strain name**	**Fusion** **cleavage** **site**	**Genotype**	**Country**
EF565077	Blue winged teal/US(LA)/88-304/1988	GERQERL	1	USA
EF565074	Green winged teal/US(LA)/88-35/1988	GERQERL	1	USA
EF565014	Chicken/US(NY)/13828/1995	GERQERL	1	USA
EF564958	Mallard/US(MD)/02-224/2002	GERQERL	2	USA
EF565017	Mallard/US(MN)/98-49/1998	GERQERL	2	USA
EF564962	Wood duck/US(OH)/02-677/2002	GERQERL	2	USA
EF564955	Mallard/US(MD)/02-195/2002	GERQERL	2	USA
EU547751	KR/duck/01/06	GERQERL	2	Korea
EF027144	Poultry/Hong Kong/1252.8/2005	GERQERL	3	Hong Kong
EF027142	Chicken/Hong Kong/1250.2/2005	GERQERL	3	Hong Kong
EF564960	Mallard/US(MD)/02-308/2002	GERQERL	4	USA
EF564966	Mallard/US(MD)/02-868/2002	GERQERL	4	USA
EF564994	Black duck/US(MD)/01-431/2001	GERQERL	5	USA
EF565023	Mallard/US(MN)/00-470/2000	GERQERL	5	USA
EF565079	Mallard/US(MN)/99-348/1999	GERQERL	5	USA
EF565065	Environment/US(NJ)/378106-4/2005	GERQERL	6	USA
EF565035	Mallard/US(MN)/00-66/2000	GERQERL	7	USA
EF564895	Mallard/US(MD)/04-118/2004	GERQERL	7	USA
EF564892	Ruddy turnstone/US(DE)/1485/2002	GERQERL	8	USA
EF565031	Blue winged teal/US(TX)/02-40/2002	GERQERL	9	USA
EF565022	Mallard/US(MN)/00-185/2000	GERQERL	9	USA

**Table 3:** Previously published F gene sequences of class II Newcastle disease virus used for phylogenetic analysis.

**GenBank** **accession** **number**	**Strain name**	**Fusion** **cleavage** **site**	**Genotype**	**Country**
AY175726	AV 963/98 NZ5/97 (GNZDK98025)	GGKQGRL	I	Ireland
AY562991	Chicken/N. Ireland/Ulster/67	GGKQGRL	I	Ireland
DQ439947	NDV05-095	GGKQGRL	I	China
EF564821	Mallard/US(MD)/04-204/2004	GGKQGRL	I	USA
EF564901	Mallard/US(MD)/04-235/2004	GGKQGRL	I	USA
EF564942	Mallard/US(MD)/04-483/2004	GGKQGRL	I	USA
AY130861	Chicken/U.S.(PA)/31003/92	GGKQGRL	I	USA
AF217084	Queensland V4	GGKQGRL	I	Australia
AY175736	AV 80/97 D813-2 (HTWDK95193)	GGKQGRL	Ia	USA
AY427817	Heb02	GGKQGRL	Ia	China
AY935489	01-1108	GRRQGRL	Ia	Australia
AY289000	Chicken/USA/Roakin/48	GRRQKRF	II	USA
AY845400	LaSota	GGRQGRL	II	China
M24695	BI/47	GGRQGRL	II	USA
AY727881	32C/T.98	GGKQGRL	IIa	Argentina
EF564834	Blue winged teal/US(LA)/87-155/1987	GGKQGRL	IIa	USA
EF564836	Blue winged teal/US(LA)/87-190/1987	GGKQGRL	IIa	USA
EF564841	Blue winged teal/US(LA)/87-247_b/1987	GGKQGRL	IIa	USA
EF564972	Mallard/US(MD)/03-152/2003	GEKQGRL	IIa	USA
EF564993	Mallard/US(MD)/03-807/2003	GEKQGRL	IIa	USA
EF565012	Mallard/US(MD)/01-618/2001	GEKQGRL	IIa	USA
EF565019	Mallard/US(MN)/98-350/1998	GEKQGRL	IIa	USA
EF565032	Mallard/US(MN)/99-397/1999	GEKQGRL	IIa	USA
EU547752	KR/duck/02/06	GGKQGRL	II	Korea
EU547755	KR/duck/05/07	GGKQGRL	II	Korea
EU547757	KR/duck/07/07	GGKQGRL	II	Korea
M24700	AUS/32	GRRQKRF	III	Australia
AY741404	Herts/33	GRRQRRF	IV	USA
AY288999	Chicken/Mexico/37821/96	GRRQKRF	V	Mexico
AY562987	Gamefowl/U.S.(CA)/211472/02	GRRQKRF	V	USA
AY288996	Pigeon/Italy/1166/00	GRRQKRF	VI	Italy
AF358786	TW/2000	GRRQKRF	VII	Taiwan
AF456442	JS/5/01/Go	GRRQKRF	VII	China
AF048763	AF2240	GRRQKRF	VIII	Malaysia
AY734534	Chicken/Trenque Lauquen	GRRQKRF	VIII	Argentina
AF456435	JS/1/97/Go	GRRQKRF	IX	China

### GenBank accession numbers

The NDV sequence data were submitted to the GenBank database; the accession numbers and other details are shown in Table 1.

## Abbreviations

AGWT: American green-winged teal; AIV: avian influenza virus; APMV: avian paramyxovirus; END: exotic Newcastle disease; HA: hemagglutination; MALL: mallard; ND: Newcastle disease; NDV: Newcastle disease virus; NOPI: northern pintail; rRT-PCR: real time reverse-transcription polymerase chain reaction; RT-PCR: reverse-transcription polymerase chain reaction; VI: virus isolation; WODU: wood duck.

## Competing interests

The authors declare that they have no competing interests.

## Authors' contributions

NJ and YC contributed for RT-PCR, sequence analysis and generation of phylogenetic tree. MA and AKC performed the virus isolation in eggs. NJ and SMG drafted the manuscript. SMG coordinated overall planning and designed this study. PTR coordinated sample collection from wild birds from Minnesota, South Dakota, and North Dakota. All authors' have read and approved the final manuscript.
